# *Galleria mellonella* as a Suitable Model of Bacterial Infection: Past, Present and Future

**DOI:** 10.3389/fcimb.2021.782733

**Published:** 2021-12-22

**Authors:** Guillaume Ménard, Astrid Rouillon, Vincent Cattoir, Pierre-Yves Donnio

**Affiliations:** ^1^ Univ Rennes, CHU Rennes, INSERM, Bacterial Regulatory RNAs and Medicine (BRM), service de Bactériologie Hygiène-Hospitalière (SB2H), UMR_S 1230, Rennes, France; ^2^ Univ Rennes, INSERM, Bacterial Regulatory RNAs and Medicine (BRM), UMR_S 1230, Rennes, France

**Keywords:** *Galleria mellonella*, infection model, pathogenic bacteria, antibacterial therapies, standardization, perspectives

## Abstract

The increasing interest for *Galleria mellonella* larvae as an infection model is evidenced by the number of papers reporting its use, which increases exponentially since the early 2010s. This popularity was initially linked to limitation of conventional animal models due to financial, technical and ethical aspects. In comparison, alternative models (e.g. models using *Caenorhabditis elegans*, *Drosophila melanogaster* or *G. mellonella*) were cheap, simple to use and not limited by ethical regulation. Since then, similar results have been established with *G. mellonella* model comparatively to vertebrates, and it is more and more often used as a robust model *per se*, not only as an alternative to the murine model. This review attempts to summarize the current knowledge supporting the development of this model, both on immunological and microbiological aspects. For that, we focus on investigation of virulence and new therapies for the most important pathogenic bacteria. We also discuss points out directions for standardization, as well as recent advances and new perspectives for monitoring host-pathogen interactions.

## Introduction

Research on animal models is essential to get more information about human infections and host-pathogen interactions. Animal experimentations had been going on for a long time since Aristotle and Hippocrate were practicing animal dissections to elucidate how the human body functions (Baumans, 2005). The rise of microbiology, and Robert Koch postulates have greatly enhanced the use of animal models to demonstrate the pathogenicity of microorganisms (Falkow, 2004). Vertebrates such as murine models constitute traditional host-models for the study of human pathogens because of a high degree of similarity in metabolism, body temperature, or immune response (Cutuli et al., 2019). Currently, about 75 to 100 million vertebrates per year are employed for scientific purposes, primarily mice and rats (Bismuth et al., 2019). In recent years, voices have been raised to protest against the untimely use of animals in research (Pereira et al., 2018). As a result, regulation has become considerably tougher. The first European directive on animal protection dates back to 1986 and is routinely reviewed (Richmond, 2000). It is mainly based on the 3Rs rules: Replace, Reduce and Refine, as described by Russel and Burch (Tannenbaum and Bennett, 2015). Ethical rules require authorizations that can induce considerable delays in approval. In addition, there are other challenges facing the scientific research: working on vertebrate models needs specific training, adequate permits and equipment, animal adaptation times and had significant costs. So, the use of a large number of mammals can be challenging for financial, technical and ethical reasons (García-Lara et al., 2005). One of the outcomes is the rationalizing of the number of animals that could result in unreliable and unpredictable data. This legislation on the animal conditions and welfare is necessary, and requires the scientific community to adapt. The *in vitro* approach to examine human pathogens is not suitable and does not mimic the natural niche. It is demonstrated that the expression of virulence factors is not the same between *in vitro* growth, including media mimicking the host’s environment, and in animals or humans. This is true for *Staphylococcus aureus*, a major human pathogen, but also for other bacterial species (Szafranska et al., 2014; Chaves-Moreno et al., 2016; Deng et al., 2019; Ibberson and Whiteley, 2019). Consequently, extrapolations from *in vitro* to more complex biological conditions are therefore subject to limits of interpretation (Swearengen, 2018). As an alternative, scientists are increasingly conducting animal experiments with alternative models such as invertebrates to allow easier determination of host effects (Pereira et al., 2018). Various invertebrate models exist and are used to study host-pathogen interactions such as the fruit fly *Drosophila melanogaster*, the nematode *Caenorhabditis elegans*, and the greater wax moth *Galleria mellonella* (Nathan, 2014). These invertebrates do not have nociceptors and are thus insensitive to pain; consequently, they are no restrictive ethical rules unlike for vertebrates (Eisemann et al., 1984). Their low cost as well as their ease of use contribute to their expanding popularity (Champion et al., 2018). Although lacking an adaptive immune system, invertebrates share with vertebrates a large number of orthologic genes responsible for general functions thus explaining that their innate immune system is similar (García-Lara et al., 2005). *G. mellonella*, the most recent invertebrate model has been less studied than the others, nevertheless its use as a model for deciphering virulence and antimicrobial efficacy is increasing (Champion et al., 2016; Tsai et al., 2016; Cutuli et al., 2019; Pereira et al., 2020). It is now considered that this larvae model constitutes a suitable model for studying human pathogens. This review aims to describe the current knowledge of the *G. mellonella* model and points out directions for standardization and new perspectives for its utilization.

## The *Galleria mellonella* Biology

The insect *G. mellonella*, also called honeycomb moth or greater wax moth, is part of the Lepidopetra order, the *Pyralidae* family, and the *Galleriinae* subfamily (Kwadha et al., 2017). It is described worldwide where beekeeping is practiced, and lives naturally in beehives where it feeds on wax and pollen, causing bee galleriosis (Singkum et al., 2019). This is a typical holometabolous insect with a full metamorphosis cycle whose larval stage (caterpillars) is of interest to the scientific community as a model of infection (Tsai et al., 2016; Kwadha et al., 2017).

### Life Cycle and Anatomical Characteristics

**Figure 1:**
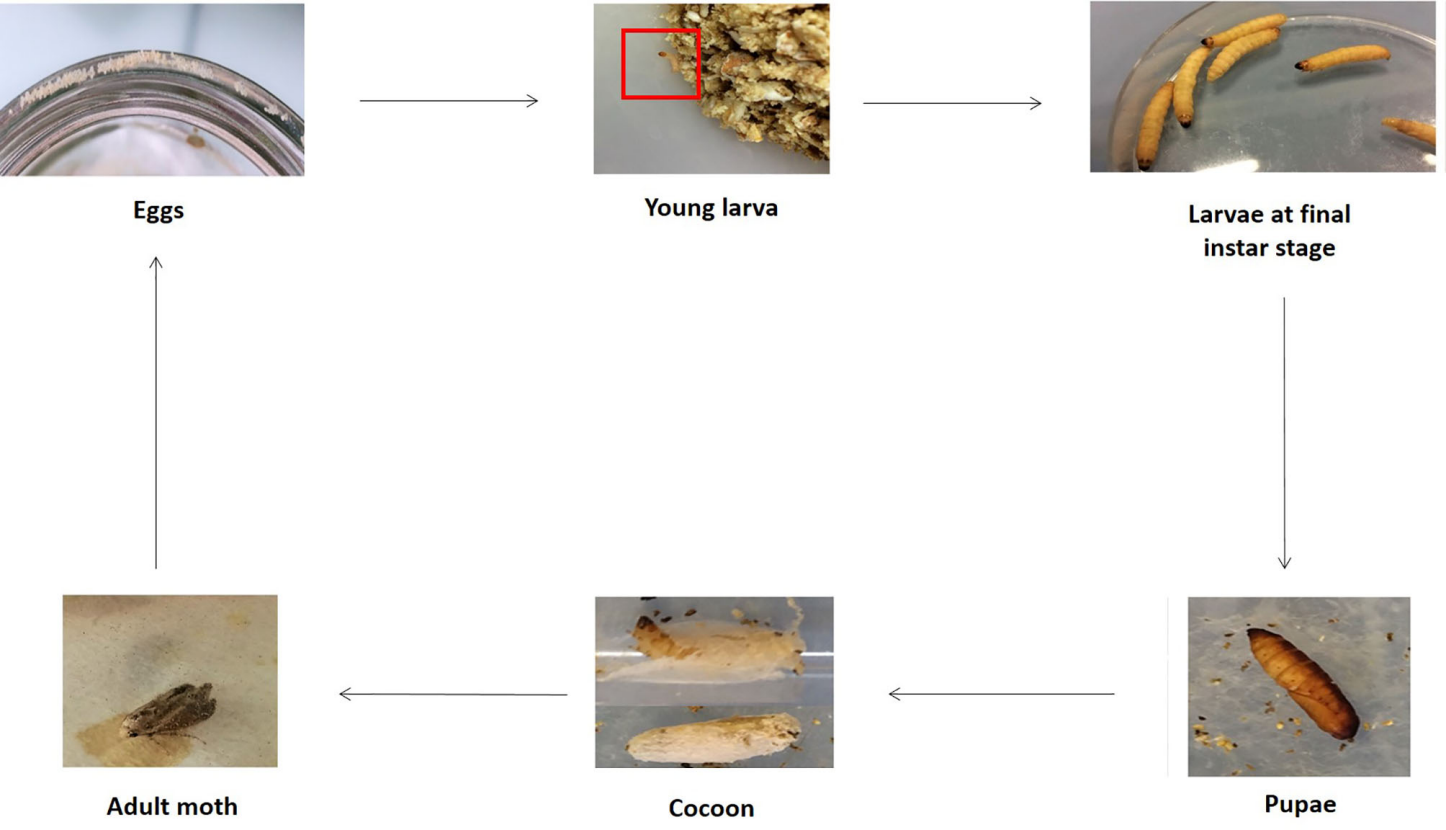
Different stages of *G. mellonella* life cycle. The fourth stages are represented e.g. eggs, larvae, pupae and adult. Larvae are visible at first molting stage and final instar stage. Cocoons are also indicated. The entire life cycle was obtained using our rearing intern protocol. (Copyright^®^: Marie Suriray).

Four distinct live stages are described: eggs, larvae, pupae and adults (**Figure 1**), with an approximate duration of a complete cycle of 8 weeks at 29-39°C and high humidity (Ellis et al., 2013). Complete metamorphosis is affected by both biotic and abiotic factors including competition for food, diet quality, cannibalism, temperature and relative humidity (Kwadha et al., 2017).

**Figure 2:**
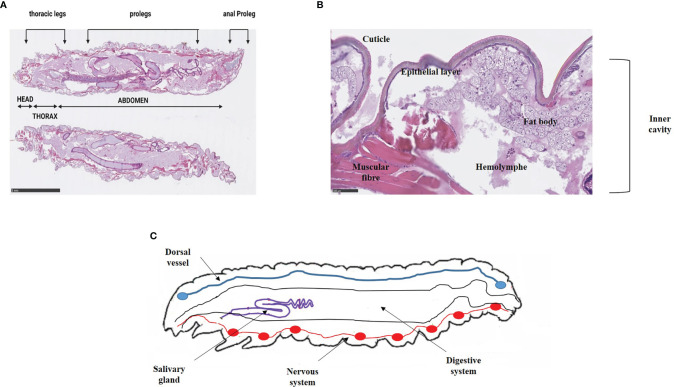
Anatomy of (*G*) *mellonella* larvae. **(A)** External anatomy of *G. mellonella* larvae. The larval body is divided into 3 distinct parts e.g. head, thorax and abdomen. Prolegs are scattered on the thorax and the abdomen. **(B)** Integument system and inner cavity. The integumentary system includes the cuticle and a thin epithelial layer opening onto the inner cavity composed of the fat body and the hemolymph. Pictures are from our immunohistochemical analysis that obtained from non-infected larvae. Scale bar is indicated (Ménard et al., 2021). **(C)** Anatomy of *G. mellonella* larvae (adapted from Durieux et al., 2021).

Initially, during oviposition, females lay 50-150 clustered-eggs whose hatching varies with temperature (Ellis et al., 2013). Indeed, there is a better development at warm temperatures than at cold ones, and eggs do not tolerate extreme temperatures. They have a spheroidal shape, and are white to light pink color. The development into larvae is temperature dependent, and last between 3 and 30 days (Charrière and Imdorf, 1999). Indeed, at a temperature comprising between 24-27°C, the duration is 3-8 days whereas at 10-16°C, the duration is about 30 days. Larvae measure 1-23 mm, are creamy-white with a reddish head and became darken as it grows. They undergo about 8-10 molting stages. The larva consists of a number of segments, divided into 3 anatomical parts: head, thorax, and abdomen. Six legs are found at the thoracic part and prolegs are present on the abdominal area (**Figure 2A**) (Wojda et al., 2020). Anatomically, the larval integumentary system comprises a thick coating called cuticle, under which is found a thin epithelial layer (**Figure 2B**). The inner cavity consisted of the fat body as well as the hemolymph, i.e. the larval circulatory system. The digestive system and the silk gland are located in the fat body (**Figure 2C**). The dorsal region, considered to be crucial by coordinating the immune response, is called the “new immune tissue” (Pereira et al., 2015; Hillyer and Pass, 2020). The ventral region corresponds to the nervous system, and consists of several ganglia (**Figure 2C**) (Singkum et al., 2019; Durieux et al., 2021). Larvae at last instar produce silk which is used to form cocoons (Singkum et al., 2019). At this moment, corresponding to the stage of pre-pupae, the larvae stop eating and become less mobile. Pupae are immobilized in cocoons and do not eat during this period. They are initially white to yellow then turn brown and finally take a dark reddish color. Adult moths color varies from reddish-brown to pale cream color. They are sensitive to light, and they live usually at night. Like the pupae stage, adult moths do not feed (Kwadha et al., 2017). Male and female adult moths have many differences. Indeed, males are slightly smaller and lighter in color than females, and they live 21 days whereas females live 12 days (Desai et al., 2019).

### Larval Microbiota Description

The development of next-generation sequencing (NGS) has led to a better understanding of microbial communities, referred as microbiota, in both humans and animals. It is now established that the microbiota constitutes an interface with the host, particularly with its immune system, and is a source of many interactions between bacteria. The *G. mellonella* larvae microbiota is poorly characterized, and few data are available. The first studies to investigate the larval microbiota described it as very simple with a main species, *Enterococcus faecalis* (Bucher and Williams, 1967). By studying the microbiome composition on various body sites of *G. mellonella* larvae, Allonsius et al. showed a predominance of *Enterococcus* species, as well as slight variation of bacterial composition of hemolymph, fat body, skin or feces (Allonsius et al., 2019). They also provided differences between two groups of larvae according their origin: bait larvae (i.e. larvae used as reptile food or fishing bait, commercially available), or research grade larvae (i.e. larvae whose rearing and storage conditions are well defined with standardized procedures). Bait larvae presented a greatest microbial diversity in the hemolymph and on the skin, probably due to previous treatments with hormones or antibiotics comparing to research grade larva. This hypothesis is in agreement with Krams et al., who report that diet diversity influence the gut microbiome composition (Krams et al., 2017). The different larval metamorphosis modified the composition of the larval digestive microbiota: more the development stage was advanced, more *Enterococcus* species were predominant, and conversely, Enterobacterales and staphylococci became undetectable (Johnston and Rolff, 2015). This is an important concept because *G. mellonella* larvae were used at the final instar-stage in virulence studies of microorganisms.

### The *Galleria mellonella* Immune System

**Figure 3:**
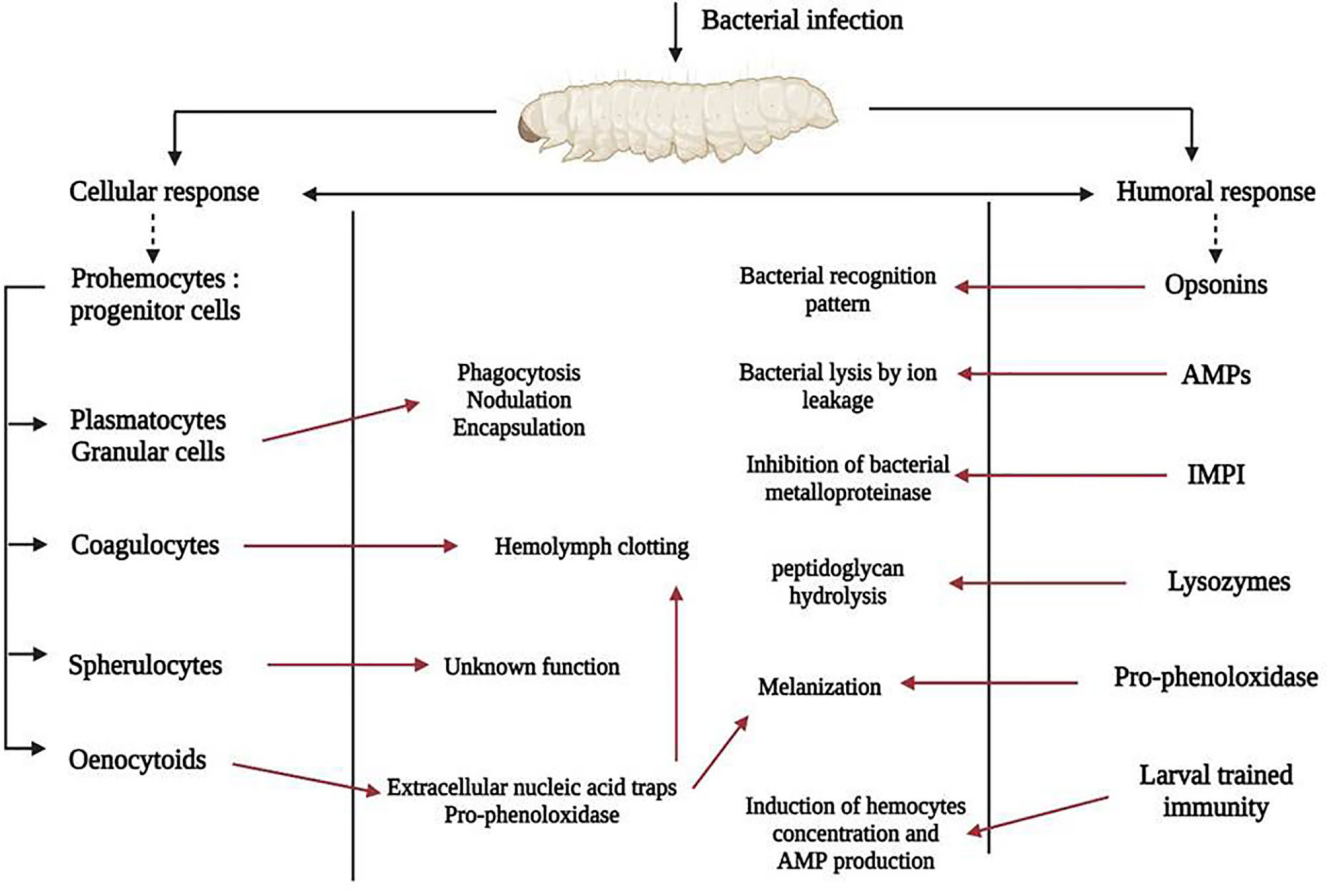
*G. mellonella* immune system activation after bacterial infection. Actors of the cellular immune response are shown on the left of the picture, those of the humoral response on the right and the pathophysiological consequences in the rectangular box. Red arrows indicate activation.

Like other insects, *G. mellonella* lacks an adaptive immune system but its innate system shares many similarities with that of mammals. It includes a cellular response in which hemocytes – immune cells close to mammal neutrophils – are key actors responsible for cellular events, and a humoral response with soluble effector molecules (**Figure 3**).

#### Cellular Immune Response

Hemocytes are predominantly found in the hemolymph, the analogue of the mammalian bloodstream, but are also subcuticular, scattered in the fat body and around the digestive tract. Hemocytes concentration fluctuates during life, and is also affected by stress caused by microorganisms (Arteaga Blanco et al., 2017). During an infection, activated hemocytes migrate to the injured site. Broadly, insects possess many types of hemocytes with particular morphological, histological and functional features (Gupta, 1985). At least, six were identified within *G. mellonella*, called prohemocytes, plasmatocytes, granular cells, coagulocytes, sphelurocytes and oenocytoids (Boman and Hultmark, 1987). They are implicated in different physiological functions: phagocytosis, nodulation, encapsulation, clotting, melanization (Boman and Hultmark, 1987; Tojo et al., 2000), and are linked to the humoral response through soluble effector molecules. Prohemocytes are progenitor cells, having the property to differentiate into several cell types (Browne et al., 2013). Plasmatocytes and granular cells are the predominant immune cells, and are key members of cellular immunity due to their role in phagocytosis, nodule formation and encapsulation (Tojo et al., 2000; Wu et al., 2016). Phagocytosis first involves granulocytes, which use opsonins to recognize microorganisms responsible for degranulation, thus allowing plasmatocytes to adhere to pathogens. Complete phagocytosis provokes killing of pathogen by mechanisms including production of reactive oxygen species (ROS) (Kavanagh and Reeves, 2004). Nodulation is mediated by the cooperation of hemocytes, which leads to the formation of several successive layers of immune cells surrounding recognized pathogens, resulting in aggregates known as nodules (Lavine and Strand, 2002). Next step is activation of the melanization process inside the nodules, responsible for death of foreign bodies (Gillespie et al., 1997). Encapsulation concerns large microorganisms such as nematodes or protozoa, but is not reported for bacteria (Boman and Hultmark, 1987). Encapsulation process involves granular cells and plasmatocytes that interact in sequential steps: granular cells recognize and attach to microorganisms leading to the release of specific peptides that then attract plasmatocytes. This process leads to the formation of superimposed hemocyte cells surrounding pathogens, which are destroyed by the release of specific molecules by the two cell types (Kavanagh and Reeves, 2004). Coagulocytes are involved in the hemolymph coagulation (clotting) corresponding to the first line of defense after exposure to a pathogen (Trevijano-Contador and Zaragoza, 2018). Spherulocytes corresponding to non-adhesive cells that transport and secrete several cuticular components but their function is still little explored (Browne et al., 2013; Arteaga Blanco et al., 2017). Oenocytoids contain and secrete precursors of the phenoloxidase, the pro-phenoloxidase, and are involved in the melanization pathway (Söderhäll and Cerenius, 1998; Lavine and Strand, 2002; Strand, 2008). In addition, like mammal neutrophils, they are able to secrete extracellular nucleic acid traps which are involved in microorganism sequestration and coagulation activation (Altincicek et al., 2008).

#### Humoral Immune Response

##### Opsonins

*G. mellonella* produces many pathogen-associated molecules opsonins that target bacteria cell-wall components such as lipopolysaccharide (LPS) or lipoteichoic acid (LTA). At least, four opsonins classes are described: apolipophorin-III (apoLp-III), peptidoglycan recognition proteins (PRGPs), cationic protein 8 (GmCP8) and hemolin (Tsai et al., 2016; Trevijano-Contador and Zaragoza, 2018). ApoLp-III, a molecule involved in the lipid transport, is the most characterized, and acts as a pleiotropic effector of larval humoral immunity. ApoLp-III is both engaged in the production of ROS and promotes the synthesis of antimicrobial peptides (Niere et al., 1999; Park et al., 2005). In insects, PRGPs are involved in the hydrolysis of bacterial peptidoglycan. Six of them were identified in *G. mellonella* using transcriptomic data, but their specific functions are not elucidated yet (Vogel et al., 2011; Tsai et al., 2016). Similarly, others opsonins such as GmCP8 and hemolin are known to bind LPS or LTA, but their functions are unclarified to date (Ladendorff and Kanost, 1990; Yu et al., 2002; Kim et al., 2010). Nevertheless, hemolin concentration in hemolymph increases during *S. aureus* infection, suggesting a putative role in immunity (Sheehan et al., 2019).

##### Antimicrobials Peptides

Antimicrobials peptides (AMPs) are ubiquitous components that play a major role in *G. mellonella* innate immunity. AMPs repertory consists of 20 peptides, at least, whose the common feature is to possess a broad spectral microbial activity (Brown et al., 2009; Vogel et al., 2011). They are found in hemocytes, fat body, salivary glands, reproductive and digestive tracts, at different concentrations both in non-infected and infected larvae (Tsai et al., 2016). Two types of AMPs are described, namely anionic and cationic antimicrobials (Trevijano-Contador and Zaragoza, 2018).

Cationic peptides are the most characterized and the main AMPs found within *G. mellonella*. They are divided into 3 groups according to their structure: linear α−helical peptides (e.g. cepropins, moricins), peptides with disulfide bridges (e.g. gallerimycin, galiomycin) and peptides with proline and/or glycine residues (e.g. Gm proline-rich peptide 1, gloverin) (Brown et al., 2009; Vogel et al., 2011; Tsai et al., 2016). AMPs actions result in a unique effect: the induction bacterial cells lysis by ion leakage, involving different mechanisms. For example, cecropins and moricins lead to the formation of cytoplasmic membrane pores whereas gloverin or Gm proline-rich peptide increase membrane permeability by blocking the synthesis of vital membrane proteins (Kim et al., 2004; Cytryńska et al., 2007; Kawaoka et al., 2008). As a common characteristic, they exhibit a broad antibacterial spectrum, even if some of them act preferentially on Gram-negative or Gram-positive bacteria or on filamentous fungi (Wojda, 2017). These observations are in agreement with the specific activation of the larval immune in response to a determined pathogen.

Anionic peptide 1 and 2 (AP1, AP2) are to date the only known member of anionic AMPs (Sowa-Jasiłek et al., 2020). AP2 is found in the hemolymph of unstimulated larvae as well as infected larvae at a high and constant level, and possesses a low activity against *Micrococcus luteus* and yeasts (Mak et al., 2010; Sowa-Jasiłek et al., 2020).

##### Insect Metalloproteinase Inhibitor

Many human pathogens secrete a wide range of metalloproteinases especially thermolysin-like metalloproteinases from the M4 family, which cleave proteins implicated in the immune response. *G. mellonella* produces an insect metalloproteinase inhibitor (IMPI) that is the only microbial-induced metalloproteinase inhibitor identified to date. IMPI can inhibit thermolysin-like metalloproteinases, and contributes to the innate immune response by counteracting secreted virulence factors (Vilcinskas and Wedde, 2002; Vogel et al., 2011; Wojda, 2017).

##### Lysozymes

Lysosymes were the first antimicrobial proteins that have been described from *Galleria* (Mohrig and Messner, 1968). They constitute a family of muramidase-like proteins able to hydrolyze strongly the peptidoglycan, of Gram-positive bacteria but moderately in Gram-negative bacteria (Yu et al., 2002; Vogel et al., 2011). Nevertheless, they act synergistically with others opsonins such as apoLp-III, leading to an increase of damages in *E. coli* (Zdybicka-Barabas et al., 2013). Lysozymes are present in hemolymph of non-infected larvae and their concentration increase in the presence of foreign bodies (Wojda, 2017; Sheehan et al., 2019). They modulate the larval microbiota since a significant increase of Enterobacterales is observed in larvae knocked-down for lysozyme production (Johnston and Rolff, 2015).

##### Melanization

During the infection process, soluble effectors molecules bind LPS or LTA bacterial components, inducing the release of pro-phenoloxidase by oenocytoids that are then activated in phenoloxidase (PO) through the serine protease cascade (Cerenius et al., 2008). PO oxidizes the phenolic compounds into quinones that are then metabolized into melanin, explaining the black spots in infected larvae (Kopácek et al., 1995). Melanization pathway participates in the antimicrobial activity against bacteria or fungi (Hoffmann et al., 1996; Jorjão et al., 2018; Pereira et al., 2018). The degree of melanization seems to be a dependent on both strain virulence and inoculum. On one hand, enteroaggregative *E. coli* triggered the melanization process while non-pathogenic *E. coli* did not (Guerrieri et al., 2019). On the other hand, larvae infected with a *S. aureus* strain at 10^6^ UFC were rapidly and totally melanized whereas with 10^4^ UFC, there is no sign of melanization (Ménard et al., 2021).

##### Larval Trained Immunity

Since invertebrates are deprived of T and B lymphocytes and do not produce antibodies, the lack of adaptive immunity apparently limits the relevance of this model to study microorganism virulence. Nevertheless, a specific response called ‘trained immunity’ has been described, which consists of an infection containment mediated by hemocytes and AMPs (Melillo et al., 2018). This mechanism is evidenced by the infection at sub-lethal concentrations followed by exposure to higher concentrations of the pathogen, resulting in more resistant larvae (Pham et al., 2007). This immune-like memory has not been clearly elucidated yet but it demonstrates the complexity of the immune response to foreign microorganisms. In *G. mellonella*, this mechanism has been described in particular in larvae-fungi interactions (Pereira et al., 2018; Trevijano-Contador and Zaragoza, 2018). The two most described AMPs, gallerimycin and galiomycin, play a role in this pre-immunization since it was shown that their concentrations increased after stimulation with a non-lethal dose of *Candida albicans* (Bergin et al., 2006). It was also demonstrated that pre-immunization of *G. mellonella* larvae with LPS or heat-killed *Listeria monocytogenes* enhanced larval survival by pre-immune activation inducing an increase of hemolymph antimicrobials (Mukherjee et al., 2010). Results were similar when larvae were pre-immunized and infected with *Klebsiella pneumoniae* (Insua et al., 2013). Infection by 10^5^ CFU of *S. aureus*, but not 10^4^ CFU, induces melanization and death (Sheehan et al., 2019). This tolerance effect would be related to primary immunization by staphylococci as recently confirmed (Sheehan et al., 2021).

## The *Galleria mellonella* Model for the Study of Bacteria of Medical Interest

### *Galleria mellonella* Larvae as a Model to Study Bacterial Virulence

Here, we describe and discuss several studies implying the *G. mellonella* model as an alternative infection model, focusing on relevant human pathogen bacteria. Frequently, this larvae model is used as a screening model because of multiple advantages described above. For example, to test the hypothesis that a gene or a system contributes to virulence, research teams monitor larval mortality over time with both the wild type strain and the mutated one, allowing for fast results (Purves et al., 2010; Quiblier et al., 2013; Lo Sciuto et al., 2018). Our objective is using methods currently available, to focus on host pathogen interactions in the *G. mellonella* model. Virulence assessment can be performed according to several approaches: clinical observations, bacterial burden, immune response activation through AMP expression, hemocyte density, phenoloxidase activity, histopathological data and monitoring of bacterial gene expression (**Table 1**).

**Table 1:** Selected studies focusing on bacteria-G. mellonella interactions throw different virulence assessments.

Bacteria	Virulence assessment	References
***L. pneumophila***	Clinical observations Histopathological data Bacterial burden Immune response activation (AMP expression)	Harding et al., 2012 Harding et al., 2013
***L. monocytogenes***	Clinical observations Histopathological data Bacterial burden Immune response activation (AMP expression, and other markers) Analyse of host miRNA	Mukherjee et al., 2010 Mukherjee et al., 2013 Mannala et al., 2017
***B. cereus***	Clinical observations Histopathological data Bacterial burden	Ramarao et al., 2012 Salamitou et al., 2000
***Shigella spp***	Clinical observations Histopathological data Bacterial burden Immune response activation (hemocytes quantification)	Barnoy et al., 2017
***C. jejuni***	Clinical observations Histopathological data Bacterial burden	Senior et al., 2011
***Y. enterocolitica***	Clinical observations Bacterial burden	Alenizi et al., 2016
***S. pneumoniae***	Clinical observations Bacterial burden Immune response activation (hemocytes quantification, detection of oxygen free radicals)	Cools et al., 2019
***L. monocytogenes***	Clinical observations Histopathological data Bacterial burden Immune response activation (hemocytes viability, phenoloxidase activity, AMP expression) Monitoring bacterial gene expression (bioluminescence)	Joyce and Gahan, 2010
***K. pneumoniae***	Clinical observations Histopathological data Bacterial burden Immune response activation (hemocytes quantification, phenoloxidase activity) Monitoring bacterial gene expression (bioluminescence)	Insua et al., 2013
***P. aeruginosa***	Clinical observations Histopathological data Monitoring bacterial gene expression (qRTPCR)	Moya-Andérico et al., 2020
***S. aureus***	Clinical observations Histopathological data Bacterial burden Monitoring bacterial gene expression (qRTPCR)	Ménard et al., 2021
***S. aureus*** ***P. aeruginosa*** ***A. baumanii***	Clinical observations Bacterial burden	Maslova et al., 2020

#### Use of the Model for Study of Intracellular Bacteria

Deciphering of intracellular bacteria interactions with the host is a major part of the acquisition of knowledge on virulence. The *G. mellonella* model was assessed to investigate these interactions. Consistencies between mammalian models and *G. mellonella* were observed with *Legionella pneumophila* infected larvae (Harding et al., 2012). The system Dot/Icm type-4 secretion system (T4SS) responsible for the translocation of proteins into host cells was determinant for the pathogenesis because of there was no mortality with a T4SS-deficient Δ*dotA* mutant. Indeed, no internalized bacteria were visualized and the mutant was quickly cleared with no replication. In contrast, infection caused by wild-type strain resulted in a rapid proliferation, an activation of the immune response implying hemocytes aggregates and nodule formation, an intra-hemocyte survival and bacterial multiplication within vacuoles. One of the effectors of the Dot translocation system, SdhA, was also crucial for the virulence since a knockout strain was rapidly killed resulting in reduced larvae mortality (Harding et al., 2013). These results were then confirmed in a murine lung infection model. The *G. mellonella* model was also shown to be a reliable surrogate model for the virulence study from another intracellular bacterium, *L. monocytogenes*. Authors demonstrated similarities in host*-L. monocytogenes* interactions between the *G. mellonella* model and vertebrates: intracellular bacteria were detected, larval mortality was correlated with bacterial growth, and the pathogenicity island *vgc* encoding the major virulence determinants was essential for the successful of the infection (Mukherjee et al., 2010). Host-pathogen interactions were also highlighted by a rapid activation of immune-responsive genes such as gallerimycin and IMPI. Same authors revealed that this bacterium can infect brain larvae with evidence of melanization containing bacteria in the cerebral tissue (Mukherjee et al., 2013). Furthermore, they identified an activation of stress and neuronal repair markers following the infection thus suggesting a connection between host-response and brain damage. In the last years, involvement of microRNAs (miRNAs) into host-bacteria interactions had emerged, and miRNA profile expression pattern could be altered during an infection, resulting in a different expression pathway of targeted mRNAs that regulate the host immune response. In the *G. mellonella* host, *L. monocytogenes* was able to modify miRNA expression with at least a different expression profile for 90 miRNAs of which mRNA-targets were identified for some of them (Mannala et al., 2017). Some mRNA targets participate to the larvae defense response thus indicating a modulation of the immune response. For example, the miRNA miR-133 was downregulated after *L. monocytogenes* infection leading to an upregulation of its target, the MAP kinase system allowing an activation of the insect prophenoloxidase cascade.

#### Use of the Model for Study of Enteric Bacteria

*G. mellonella* is also an alternative model to investigate enteric bacteria pathogens. Indeed, similarities are described between intestinal epithelial cells from larvae and from mammalian digestive tract (Ramarao et al., 2012). By an oral infection to respect the natural route of contamination, with a mixture of *Bacillus cereus* spores or vegetative cells and toxins, a synergistic effect was observed, mimicking a gastrointestinal infection (Salamitou et al., 2000). These results highlighted that spores resisted to stress induced by host larvae and could turn into vegetative bacteria explaining the increase of the bacterial count observed. Pathogenicity would be explained by the action of the toxin, enhancing the multiplication of bacteria in the gut, which then spread by breaking the intestinal barrier (Salamitou et al., 2000; Ramarao et al., 2012). Others enteric bacteria pathogens were tested in this model such as *Yersinia pseudotuberculosis*, *Campylobacter jejuni, Yersinia enterocolitica, Shigella* spp. (Champion et al., 2009; Senior et al., 2011; Alenizi et al., 2016; Barnoy et al., 2017). Surprisingly, the majority of studies did not practice oral infection but systemic infection by injected larvae with a standardized inoculum. Barnoy et al. testing the *Shigella* virulence by oral force feeding observed no clinical larval manifestations whereas larvae died quickly after injection (Barnoy et al., 2017). Results were similar with *C. jejuni* or *Y. pseudotuberculosis* infected larvae, which were also competent for an intra-hemocyte survival (Champion et al., 2009; Senior et al., 2011).

#### Interplay Between Hemocytes and Bacteria

All the studies described above prove an activation of the larval immune system regardless of the pathogen of interest. Some have shown a decrease in hemocyte count after infection (Insua et al., 2013; Barnoy et al., 2017) but the fate of hemocytes is unknown, particularly for morphological and/or ultrastructural modifications or protein markers. This issue has been explored through the interaction between *P. aeruginosa* and hemocytes (Mizerska-Dudka and Andrejko, 2014). Eighteen hours after the infection, hemocyte modifications were detected including swollen or naked nucleus, damaged organelles and condensed chromatin. All these alterations increased over time. LCB3 protein and caspase were detected, indicating the progression of hemocytes towards a programmed cell death or apoptosis. Conversely, no hemocyte variation was observed after a *Streptococcus pneumoniae* infection with larvae-killing doses and increased intra-hemocyte production of oxygen free radicals (Cools et al., 2019). Since this increase should lead to apoptosis and necrosis, a lower number should be expected. Authors hypothesized a steady-state between the production of hemocytes and their destruction by bacteria. Interplay between *K. pneumoniae* and larvae pointed out the immune response modulation for the benefit of bacteria that enabled coexistence within the host (Insua et al., 2013). Infection was followed by bacterial proliferation, signs of hemocyte damages, and decrease of hemocytes number. Curiously, no intra-hemocyte *K. pneumoniae* were detected, and the production of AMP was not activated. The same observation in experimental *K. pneumoniae* pneumonia in mouse macrophages, suggests an escape way to survive in the host.

#### Monitoring Bacterial Virulence Factors: A Novel Aspect for Host Pathogen-Interaction

Available methods and techniques as well as increasing knowledge on this infection model are converging towards for more sophisticated studies revealing the complete potential of the greater wax moth. Larvae display intrinsic autofluorescence, which limits the use of fluorescent proteins to monitor the evolution of the infection over time, so this challenge was resolved by using bacterial gene bioluminescence and/or bacterial RNA expression. Using bioluminescence, the expression of the *L. monocytogenes* major virulence factors was measured, revealing a significant induction of temperature-dependent gene expression (Joyce and Gahan, 2010). The *K. pneumoniae* capsule polysaccharide (CPS) is an important virulence factor in the pathogenesis of pneumonia and urinary tract infections, the *Galleria* model was used to support these facts by mimicking a systemic infection (Insua et al., 2013). The CPS has been proved crucial also in invertebrates as larvae lethality was decreased with a Δ*cps* strain and associated with a reduced bacterial survival after 24h. The monitoring of *cps* bioluminescence expression levels and genes encoded lipid A remodeling validates these findings, and revealed an expression peak during the first hours of infection. Bioluminescence combined to bacterial RNA expression highlighted the importance of ribonucleotide reductases (RNR) by monitoring the expression of genes encoded for these RNR toxins in *Pseudomas aeruginosa* infected larvae (Moya-Andérico et al., 2020). We have recently used this model to investigate the role of *S. aureus* small regulatory RNAs (sRNAs), provide evidence that the sRNA expression profile in infected larvae differs sharply from *in vitro*. sRNAs were tightly regulated at the different stages of infection (Ménard et al., 2021). Some sRNAs are linked to virulence but for the most part, their function has not been elucidated, so the *G. mellonella* host would be a reliable tool to screen their implication into virulence. Others *S. aureus* virulence factors were also *in vivo* monitored in a context of device associated infections (Mannala et al., 2021). Few genes were upregulated *in vivo* as expected such as *atl*, *icaA*, and *sarA* unlike *fib*, *fnbA* and *fnbB* that are normally involved in biofilm biogenesis. This last comment could be related to the lack of fibrin and fibronectin in *G. mellonella* larva.

#### Innovating by Implementation of Novel Infection Models

Experimental studies of infected burn wounds in mammals are unwieldy due to ethical considerations. The *G. mellonella* infection model has been tested recently for this purpose (Maslova et al., 2020). It was possible to simulate a burn wound by applying a preheated metal element to the larval surface. Spontaneous mortality was correlated to burn wound surface and fluid resuscitation e.g. injection of a saline solution. Then, infection by different pathogens, *P. aeruginosa*, *S. aureus*, and *Acinetobacter baumannii*, results in increased mortality with a bacterial dissemination. This model could therefore constitute a new alternative to analyze biofilm formation and to test compounds in this context.

Recently, the study of bacterial biofilm infections was successfully adapted *in vivo* in the *G. mellonella* model especially with *S. aureus*. Two techniques were employed to simulate *S. aureus* infection on indwelling devices: toothbrush bristles insertion and stainless steel or titanium implants (Campos-Silva et al., 2019; Mannala et al., 2021). First, abiotic surface insertions did not result in excess larval mortality, and secondly, by scanning electron microscopy, it was possible to observe *S. aureus* biofilm on indwelling devices and to determine the main steps of biofilm genesis such as attachment, proliferation and detachment. Interestingly, in all cases, larval mortality was higher in biofilm group than in non-biofilm group. The *G. mellonella* biofilm model was also used to evaluate efficacy of several antibiotics in the case of *P. aeruginosa* or *K. pneumoniae* infections (Benthall et al., 2015). Together, these information support that the *G. mellonella* model constitute a suitable alternative to study bacterial biofilm and to test antibacterial compounds.

For the first time, the *G. mellonella* model was successfully used to demonstrate the pathogenicity of the *M. tuberculosis* complex (Li et al., 2018). Unlike the murine model that does not produce normally granulomas, a pathognomonic sign of tuberculosis infection, these aggregate structures were observed both in the hemolymph and the fat body of *G. mellonella*. Authors also provided that bacteria were phagocytosed by hemocytes, multiplied inside them, and then formed granuloma during the infection. These preliminary results pave the way to others studies such as testing the responsiveness of antituberculosis drugs. Indeed, the most common classes of anti-tuberculosis drugs have recently been screened, showing a convincing efficacy of most of the drugs tested (Asai et al., 2019). Rifampicin and isoniazid were the most efficient molecules both in terms of larval mortality and reduction of bioluminescence.

### *Galleria mellonella* Larvae as a Model to Test Novel Antimicrobial Alternatives

Currently, antimicrobial resistance (AMR) is a major public health issue, and some predictions are not favorable, with a number of deaths related to AMR expected to be around 10 million per year in 2050 if the situation remains as it is (Price, 2016; Tacconelli and Pezzani, 2019). World Health Organization has listed the pathogens to be closely monitored grouped into the ESKAPE clad including *Enterococcus faecium*, *S. aureus*, *K. pneumoniae*, *Acinetobacter baumannii*, *P. aeruginosa* and *Enterobacter* spp. (Mulani et al., 2019). Therefore, it is crucial to develop alternative solutions to conventional antibiotic treatments because few antibiotic compounds have been developed in recent years. Alternative therapies such as antibiotics in combination or with adjuvants, bacteriophages, AMPs, phytochemicals, are increasingly studied (Mandal et al., 2014; Taneja and Kaur, 2016). The major advantage of this invertebrate model is to allow fast screening tests of new therapies, which will be validated subsequently in a mammalian model. The aim is to limit the untimely use of mouse models. In this respect, the *G. mellonella* model has been widely used to test the potential effectiveness of such alternative therapies (**Table 2**).

**Table 2:** Utilization of G. mellonella larvae as a screening model to test the efficacy of new antibacterial therapies.

Antibiotic combinations or with adjuvants			Bacteriophages		Phytochemical therapies		
Bacteria	Association	References	Bacteria	References	Bacteria	Products	References
***E. faecium***	Linezolid + fosfomycin	Qi et al., 2019	*E. faecium*	El Haddad et al., 2019	*S. aureus*	Cinnamaldehyde	Ferro et al., 2016
	Oritavancin + others antibiotics	Meyer et al., 2019	*S. aureus*	Tkhilaishvili et al., 2020		Myricetin	Silva et al., 2017
***S. aureus***	Linezolid + fosfomycin	Li et al., 2020	*K. pneumoniae*	Thiry et al., 2019		*Eugeria brejoensis* essential oil	Bezerra et al., 2020
***K. pneumoniae***	Ceftazidime/avibactam + others antibiotics	Nath et al., 2018	Enterobacterales	Manohar et al., 2018		Epigallocatechin gallate	Knidel et al., 2019
***E. cloacae***	Imipenem + colistin	Yang et al., 2018	*P. aeruginosa*	Jeon and Yong, 2019			
***A.Baumannii***	urea-derived compound + colistin	Minrovic et al., 2018					

#### Antibiotic Combinations or With Adjuvants

Association of linezolid plus fosfomycin was statistically more effective with an enhanced survival rate than linezolid alone against vancomycin-resistant *E. faecium* (VRE)-infected larvae (Qi et al., 2019). However, there were no difference between the combination treatment group and the use of fosfomycin alone. Oritavancin, a new glycopeptide vancomycin-derivative antibiotic, with an extended half-life span, has been successfully tested in the *G. mellonella* model after infection of both vancomycin-susceptible and vancomycin-resistant strains (Meyer et al., 2019). Results with oritavancin were superior to other antibiotics alone or in combination (ceftriaxone, gentamicin and daptomycin), and no two-drug regimens including oritavancin has shown superiority over oritavancin alone.

Antibiotic combinations were also tested on *S. aureus* infected larvae. The double-therapy linezolid plus fosfomycin even at low-dose was shown to act synergistically, reduce mortality and hemolymph bacterial burden (Li et al., 2020). The fact that it can be used in low doses is of real benefit in limiting side effects (hematological cytotoxicity) and the appearance of resistant mutants.

Extended-spectrum β-lactamase (ESBL)- or carbapenemase-producing Enterobacterales are one of the most important challenges in global health due to multidrug-resistance and worldwide spread. Ceftazidime/avibactam alone at high doses, or combined with other antibiotics (polymyxin B, amikacin or meropenem) reduced significantly larvae mortality after infection by KPC-producing *K. pneumoniae* strains resistant to ceftazidime/avibactam (Nath et al., 2018). Combination of ceftazidime/avibactam and meropenem seems to be the most promising based on toxicity evidence and low potential of resistance emergence.

After establishing that the *G. mellonella* model was an accurate model to investigate *E. cloacae* virulence, Yang et al. shown that the association imipenem-colistin provided some interesting results in this invertebrate model, and could be a potential alternative when bacteria were pan-resistant (Yang et al., 2017; Yang et al., 2018).

For multidrug-resistant (MDR) opportunistic pathogens such as *A. baumannii* or *P. aeruginosa*, the *G. mellonella* model was successfully used as a screening model for alternative therapies. It was demonstrated that a urea-derived compound used as an adjuvant potentiated colistin action both *in vitro* and *in vivo* in a *G. mellonella* model infected by a MDR *A. baumannii* strain (Minrovic et al., 2018). Indeed, combination of colistin plus adjuvant compared to colistin alone or other antibiotics enhanced larvae survival. These preliminary results imply a lower colistin dosage and consequently associated renal and neurological toxicities, and suggest a colistin activity restauration on resistant strains by the adjuvant which would block resistance mechanisms.

The new multifunctional adjuvant, the non-ribosomal tobramycin-cyclam conjugate, could change therapeutic deadlock situations since it significantly restored both meropenem or aztreonam or ceftazidim/avibactam activity, and resulted in prolonged larvae survival infected with MDR *P. aeruginosa* strain (Idowu et al., 2019).

As previously explained, AMP role is crucial in larvae immune humoral response. Eisenhardt et al. highlighted that IMPI from *G. mellonella* inhibited the *in vitro* elastase and the secretome activity of *P. aeruginosa* strains, and confirmed these results in an *in vivo* wound model (Eisenhardt et al., 2019). Insect AMP such as IMPI could be another strategy to treat bacterial infections. In that sense, cecropin A2 from the insect *Aedes aegypti* was shown to attenuate *P. aeruginosa* infected larvae mortality when in combination with tetracycline (Zheng et al., 2017).

#### Bacteriophages

Using a cocktail of specific bacteriophages against VRE-infected larvae El Haddad et al. demonstrated an improving survival rate of infected larvae compared to the phage-free group (El Haddad et al., 2019).

Two types of bacteriophages, Staphylococcal bacteriophage and PYO bacteriophage respectively could both treat and prevention Methicillin resistant *S. aureus* (MRSA) infection (Tkhilaishvili et al., 2020). Indeed, at therapeutic doses, phages presented no larval toxicity, reduced larvae mortality rates when administrated before and after infection. The effect was dose-dependent and increased survival rates were observed with phage cocktails.

Thiry et al. determined that new bacteriophages at low doses were effective against KPC-producing *K. pneumonia* (Thiry et al., 2019). However, authors conclude that although the *G. mellonella* host-model is a reliable tool for fast results, it would be premature to predict the effect on mammals requiring higher doses of bacteriophages. Others found the same results by examining phage effects on several carbapenem-resistant Enterobacterales (Manohar et al., 2018): phage cocktails reduced larval mortality by 90% with a single dose, a figure that reached 100% with three successive doses.

Similar results were also obtained on MDR or extensively drug-resistant *P. aeruginosa* strains with the use of bacteriophages or adjuvants respectively (Jeon and Yong, 2019). Interestingly, the two novel bacteriophage effects were validated first using the *G. mellonella* host and then in a mouse acute pneumonia model thereby exhibiting the robustness of this invertebrate model.

#### Phytochemical Therapies

Phytochemical therapies have emerged in recent years as a potential alternative to conventional antibiotics. These anti-virulence therapies would reduce bacterial adaptation by acting on multiple targets. For example, *Eugeria brejoensis* essential oil inhibits Staphyloxanthin production in *S. aureus* and acts synergistically with conventional antibiotics (Bezerra Filho et al., 2020) and Myricetin downregulates multiple *S. aureus* virulence factors involved in adhesion, biofilm formation or hemolysis (Silva et al., 2017). Numerous plant-derived products have been evaluated in the *G. mellonella* model, in particular to target *S. aureus*, whether they are phenolic or flavonoid compounds (myricetin, epigallocatechin gallate) or molecules derived from plant oils (cinnamon oil, *Eugeria brejoensis* essential oil) (Ferro et al., 2016; Silva et al., 2017; Knidel et al., 2019; Bezerra Filho et al., 2020). All showed reduced larvae mortality with no toxicological effect when infected by *S. aureus*, so they might be promising substances to treat *S. aureus* infection including Methicillin-resistant strains.

Combination of two polyphenols, theaflavin and epicatechin was effective on MDR *A. baumannii* infected larvae as recently shown (Betts et al., 2017). These two bioactive compounds act synergistically and have a bactericidal effect resulting in increased larvae survival when compared to their use alone.

### Relevance of *Galleria mellonella* Infection Model Among Other Invertebrate Models

Traditionally, two other invertebrate models are widely described to study the virulence of bacteria or to test the effect of antimicrobial compounds: *C. elegans* and *D. melanogaster*. The *G. mellonella* model has several advantages over these two (**Table 3**). It is established that the temperature influences the behavior of bacteria because the expression of many genes is under the control of temperature (Konkel and Tilly, 2000). For example, the *L. monocytogenes prfA* regulon regulates many virulence factors thus allowing bacteria adaptation to this host (Freitag et al., 2009). *prfA* expression is a function of temperature: at 20°C, it is not activated and becomes so at 37°C, *i.e.* the temperature of the human body. By comparing the expression of the *prfA* regulon and associated virulence determinants at both 37°C and 30°C in the *Galleria* model, it was shown that these factors were more weakly expressed at 30°C than at 37°C (Joyce and Gahan, 2010). *C. elegans* and *D. melanogaster* models cannot withstand temperature of 37°C whereas *G. mellonella* does, a considerable benefit for investigating major human pathogens (Desalermos et al., 2012). Furthermore, it was demonstrated that incubation temperature induced different *G. mellonella* larval mortality profiles by studying virulence group A streptococci and *Shigella* spp. (Loh et al., 2013; Barnoy et al., 2017). These results indicated that temperature requirements are an unavoidable prerequisite to consider host-pathogen interactions rigorously. Another important consideration is the immune cell inability of *C. elegans* to phagocytize microorganisms unlike *G. mellonella* that exhibits professional phagocytes conferring it a major advantage for the study of pyogenic bacteria such as *S. aureus*, *P. aeruginosa* or *S. pneumoniae* (Champion et al., 2009; Desalermos et al., 2012). In the *C. elegans* model, bacteria proliferate in the intestinal lumen but do not penetrate intestinal epithelial cells, making it difficult to study the virulence of intracellular bacteria such as *L. pneumophila* (Harding et al., 2012).

**Table 3:** Main advantages and inconvenient among 3 major invertebrate models.

Models	Comparisons between major invertebrates models		
	*G. mellonella*	*D. melanogaster*	*C. elegans*
**Survival at 37°C**	Yes	No	No
**Phagocytosis by immune cells**	Yes	Yes	No
**Invasion of intestinal epithelial cells by bacteria**	Yes	Yes	No
**Animal size**	Significant	Small	Small
**Stock centers**	No	Yes	Yes
**Commune databases**	No	Yes	Yes
**Standardized procedures**	No	Yes	Yes
**Mutant strains available**	No	Yes	Yes

Furthermore, a technical aspect facilitate the use of the *G. mellonella* model. Its length varies between 3-30 mm, thus significantly higher than those *D. melanogaster* and *C. elegans*, (3 mm and 1 mm, respectively), which facilitates the operation and handling of the larvae (Cutuli et al., 2019). Conversely, improvements are needed so that *G. mellonella* larvae will be widely accepted as a leading infection model. In particular, the lack of stock centers and databases such as Flybase or WormBase as well as standardized procedures or mutant strains are significant hindrance that can be explained because this model is still in its infancy (Cook and McArthur, 2013).

## Current Limitations of the *G. mellonella* Model and Areas of Improvement

No animal model is perfect (Baxter and Griffin, 2016). Ideally, to study host-pathogen relationships, one would need to work directly in humans, which is not feasible for both ethical and technical reasons. It is therefore necessary to accept a compromise by choosing the most suitable animal model on the basis of ethical, economic and scientific arguments. In this sense, the *G. mellonella* model has proven its usefulness as a screening model for the study of the virulence of many microorganisms, with results confirmed in mammalian models. However, this robust model has shortcomings that do not allow an optimal use making complex comparisons between scientists.

To date, the main limitation to the use of this model is the lack of standardized procedures, especially in the obtaining and storage conditions of the larvae. For example, previous works using *G. mellonella* as a host model for study *S. aureus* virulence or testing antibacterial agents showed many differences. Three labs purchase larvae in fishing bait stores (Purves et al., 2010; Quiblier et al., 2013; Sheehan et al., 2019), one used internal rearing protocol with artificial diet (Silva et al., 2017) whereas for two another studies there is no data available about origin of larvae (Desbois and Coote, 2011; Ferro et al., 2016). These different processes are sources of variability since it was shown that larvae from fishing bait stores or pet shops contain antibiotics or hormones that lead to variable and inconsistent results (Champion et al., 2018). It was demonstrated that different artificial diets influenced the survival of larvae after infection caused by *S. aureus*, *E. coli* or *C. albicans* (Banville et al., 2012; Jorjão et al., 2018). Diet influences immune system activation, responsible for fluctuations in hemolymph volume and hemocyte concentration. Commonly, larvae are not fed during infection experiments, and it is advised to starve larvae before infection (Ramarao et al., 2012; Tsai et al., 2016). However, since diet and immune activation are linked, it would be interesting to monitor larvae mortality with and without added food during infection process in order to observe any potential effect.

It would be useful to create stock centers for research on *G. mellonella* larvae as it exists for the *D. melanogaster* (Tsai et al., 2016). Recently, Biosystems technology Ltd developed standardized *G. mellonella* larvae called TruLarv™ that are age and weight defined and whose breeding conditions are normalized without hormones and antimicrobials (https://biosystemstechnology.com/products/trularv). Experiments with those standardized larvae delivered more suitable and reproducible results with several pathogens (Thelaus et al., 2018; Wagley et al., 2018; Kandiah et al., 2019). In addition, the use of calibrated reference populations would facilitate comparison of works from different labs.

Larvae storage conditions differ from one study to another with an impact on results. Larvae are often stored in the dark but at various temperatures and for longer or shorter periods after receipt (Peleg et al., 2009; Senior et al., 2011; Loh et al., 2013; Li et al., 2018; Six et al., 2019). The pre-incubation stage is crucial because when the duration is extended, the larvae were more susceptible to pathogens, with an altered immune response (Browne et al., 2015).

Another critical aspect is the larvae weight used in the experiments. In six previous publications, the weight ranged from 150 to 700 mg (Ba et al., 2015; Ebner et al., 2016; Ferro et al., 2016; Dong et al., 2017; Bazaid et al., 2018; Mannala et al., 2018). In 2019, a method was proposed to standardize the intra larval concentrations of bacteria after injection (Andrea et al., 2019). Authors showed a strong correlation between larvae weight and liquid volume allowing then to estimate with precision the *in vivo* concentration of bacteria and drugs administered.

**Figure 4:**
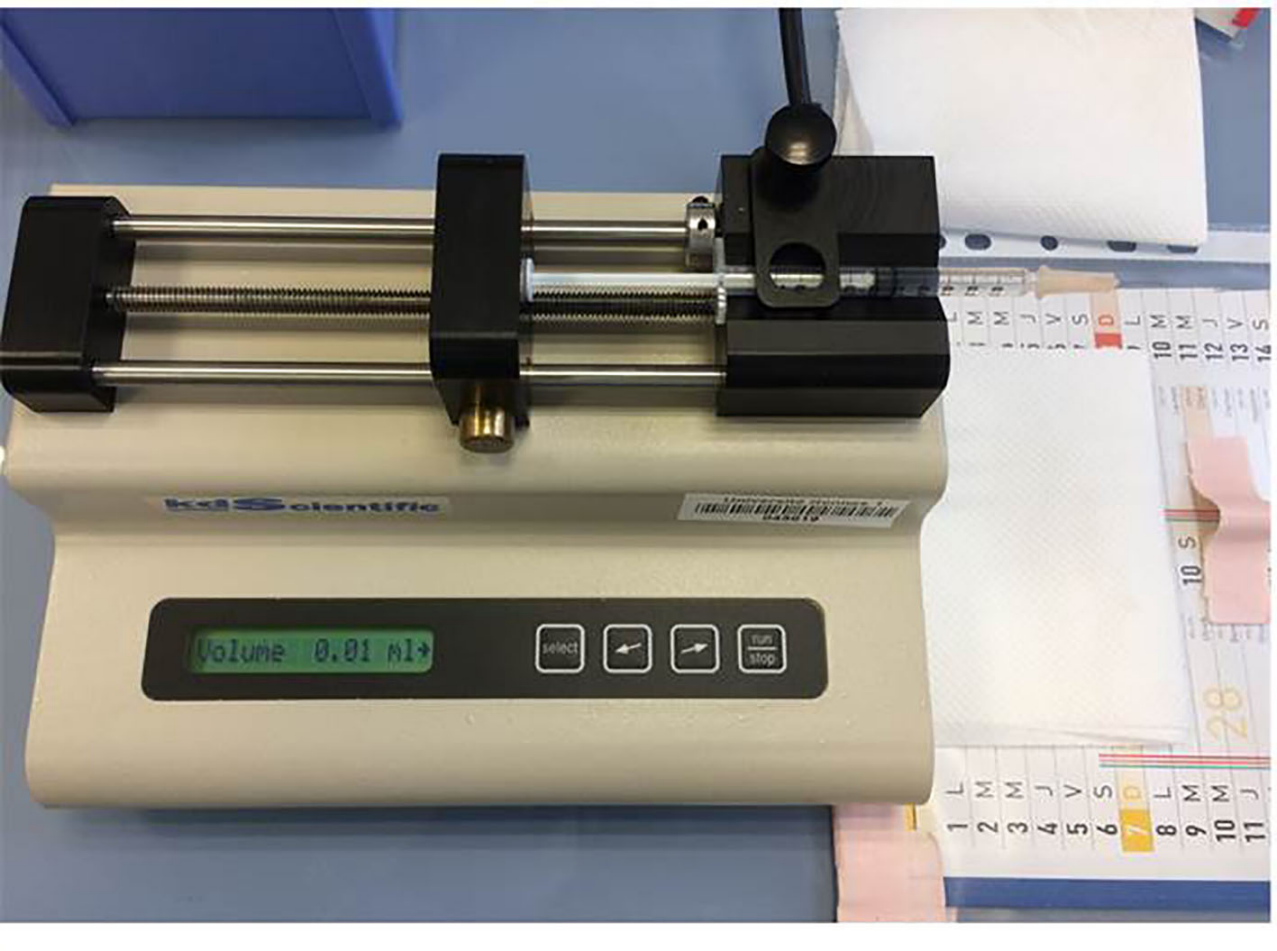
Microinjector (KDS 100 automated syringe pump, KD scientific) with a calibrated 0.3 ml tuberculin.

*G. mellonella* infections can be proceeding either by ingestion or by intra-haemocolic injection in the last proleg, this last method allowing a tight control of the inoculum using a microinjector with a calibrated syringe (**Figure 4**) (Ramarao et al., 2012). Methods for injection are not the same between laboratories (Dalton et al., 2017), and at least, three methods are described (Fuchs et al., 2010; Harding et al., 2013). The differences are not related to the number of bacteria injected but rather the time it takes to infect one larva and the risk of injury for the operator. A recent study showed that with simple restraint devices, there was a reduction of larvae manual handling with a rapid rate of injection, combined with increased protection against needle stick injury (Fredericks et al., 2020).

Considerable progresses have been made to improve interpretation of tests with this model. First studies calculated larvae mortality at the endpoint to objective the LD_50_ (Champion et al., 2018). Larvae were considered dead if they were immobile, no longer responding to stimuli and were melanized (Ramarao et al., 2012). This approach can be considered as subjective because melanization process may differ from one larva to another. A variant is to calculate a larval virulence index allowing an inter-laboratory easier comparison (Brunke et al., 2015; Ames et al., 2017). A health index scoring system dedicated to the *G. mellonella* model was also introduced to monitor severity and mortality (Loh et al., 2013). It includes 4 grades (activity, cocoon formation, melanization, and survival) and is rated from 0 to 10: the higher the score, the healthier the larvae (**Table 4**). There is still need for improvement since the weighting of the health index scoring system is sometimes imperfect. For example, larvae transformed in the pupal stage are excluded from the monitoring because it is impossible to attest melanization once the chrysalis is formed, just as when a complete cocoon surrounds the larva.

**Table 4:** Health index score system of *G. mellonella* larvae (adapted from Loh et al., 2013).

Grades	Details	Rating
**Activity**	No activity	0
	Minimal movement on stimulation	1
	Movement when stimulated	2
	Movement without stimulation	3
**Cocoon formation**	No cocoon	0
	Partial cocoon	0.5
	Full cocoon	1
**Melanization**	Black spots on brown larvae	1
	≥ 3 spots on beige larvae	2
	≤ 3 spots on beige larvae	3
	No melanization	4
**Survival**	Dead	0
	Alive	2

This score ranges from 0 (dead larva) to 10 (healthy larva) and is determined according to 4 criteria.

Over the past decade, *G. mellonella* entered the omics era, and both trancriptomic and proteomic data provided a more suitable comprehension of this model host. In 2011, the first transcriptional analysis of immune system genes was reported and numerous genes encoding immune proteins as well as effector soluble molecules were identified (Vogel et al., 2011). From this transcriptomic project, several genes were associated with immune functions after infection with *L. monocytogenes* (Mukherjee et al., 2010; Mukherjee et al., 2013). Moreover, miRNAs were also discovered and associated with the immune response after contact with human pathogens such as *E. coli* and *L. monocytogenes* (Mannala et al., 2017; Mukherjee et al., 2020). However, numerous immune proteins were still not characterized and have unknown functions. Majority of studies dedicated to observe humoral response after bacterial exposure focused on already known AMPs such as lysozyme, galliomycin, gallerimycin, cecropin (Mukherjee et al., 2010; Insua et al., 2013; Andrejko et al., 2014). Recently, a quantitative shotgun proteomics was successfully realized in response to a *S. aureus* infection demonstrating a rapid and coordinated humoral immune response (Sheehan et al., 2019). Still using a proteomic approach, these authors have mapped the proteome of both infected larvae and a fungal pathogen. By this dual approach, they were able to describe the genesis of the infection, from host-pathogen recognition to the process of melanization and encapsulation (Sheehan et al., 2020). In 2020, the Antimicrobial Peptide Database reported about 310 AMPS in insects thus suggesting that the 20 or so peptides identified in *G. mellonella* constitute only a tiny part of the humoral repertory, and that further studies are required to get more information about this humoral response (Sowa-Jasiłek et al., 2020).

At last, *G. mellonella* genome is now sequenced, but it is still not entirely analyzed and annotated (accession number: NTHM00000000) (Lange et al., 2018). The genome sequenced was obtained from the isolate FT-Tue with a total of 2,141,900 reads and 20,638,932,410 bases. Moreover, a *G. mellonella* transcriptome database is now available (https://www.uni-giessen.de/fbz/fb08/Inst/bioinformatik/Research/Supplements/galleria) (Mannala et al., 2017). These preliminary data might allow the implementation of a shared-file database from genomic, transcriptomic and proteomic projects.

## Perspectives: Advent of High-Throughput Technologies

The *G. mellonella* infection model is now well demonstrated as a screening model, enabling to study bacterial virulence by monitoring larval survival, bacterial load, immune response of infected larvae or histological data. So, the remaining question to be asked is can we go further in the study of virulence and host-pathogen interactions? High-throughput technologies (genomics, transcriptomics, proteomics or metabolomics), have revolutionized biomedical research (Hasin et al., 2017). To properly establish, survive and grow in a host, bacterial pathogens must counter hostile conditions related to host immune response and environmental conditions, and also compete with other bacteria for nutrient deprivation. This adaptation leads to an alteration in patterns of gene expression (Waddell et al., 2007; Dastghey and Otto, 2015; Reniere, 2018). In the field of infectious diseases, omics methods such as transcriptomics not only allow to notify the presence of a gene but also to know if it is expressed through a quantification of host and/or bacterial cellular mRNA transcripts (Westermann and Vogel, 2018). These high-throughput technologies are crucial from both diagnostic and therapeutic points of view, and have been extensively applied to human pathogenic bacteria both *in vitro* and *in vivo*, including humans or mammalian models (Yan et al., 2013; Szafranska et al., 2014; Westermann et al., 2017; Deng et al., 2019; Ibberson and Whiteley, 2019).

To date, few studies are available about bacterial pathogen gene expression within the *G. mellonella* host whether by bioluminescence or RT-qPCR (Joyce and Gahan, 2010; Insua et al., 2013; Moya-Andérico et al., 2020; Ménard et al., 2021; Mannala et al., 2021). The main reason is probably related to the lack of standardized procedures as previously explained. Nevertheless, the implementation of stock centers with referenced, standardized and sequenced larvae could solve this problematic, and therefore could herald the development of bacterial transcriptomic studies in this host-model.

A key step in transcriptomic studies is the quality and quantity of bacterial RNA. In most organisms, the vast majority of RNA corresponds to ribosomal RNA (rRNA) in bacteria as well as in mammals or in vertebrates (Kraus et al., 2019), and must be depleted to enable a suitable analyze of transcriptomic data which corresponds predominantly of mRNA (Culviner et al., 2020). The major issue is that several commercial kits are available to deplete rRNA from mammalian samples but none are validated to invertebrate models such as *G. mellonella*. Nonetheless, the *in vivo* transcriptome of two microorganisms (*Coxiella Burnetii* and *Yersinia entomophaga*) within *G. mellonella* system have been realized (Paulson et al., 2020; Kovacs-Simon et al., 2021). In these two studies, the authors succeeded to extract intact bacterial RNA in sufficient amount from the infected pool hemolymph. *In vivo* analysis of the transcriptome of *C. burnetti* as well as *Y. entomophaga* revealed that a significant number of genes were expressed differently, either increased or decreased compared to *in vitro* media. Pattern expression profile genes were then compared to mammalian cell lines or a mouse model, highlighting many similarities. These two pioneering studies demonstrate the *G. mellonella* larvae model relevance, no longer as a simple screening model, but also as a genuine infection model allowing to explore the virulence regulation at a transcriptomic level, and thus to better define the complex systems that are bacterial regulation networks. This novel approach deserves to be expanded for the study of major pathogenic bacteria, not only in the hemolymph but also in other larval anatomical sites, with the prerequisite of selective cell lysis to obtain bacteria-enrich samples. By combining high throughput technologies with the advantages of the *G. mellonella* model would make it possible to get more easily comprehensive bacterial transcriptomic data. Finally, to concretize these new developments, conducting dual-RNAseq would provide the opportunity to simultaneously obtain details about complex interactions host and bacteria.

## Author Contributions

GM and P-YD contributed to conception, design and writing the first drive of the manuscript. All authors contributed to manuscript revision, read, and approved the submitted version.

## Conflict of Interest

The authors declare that the research was conducted in the absence of any commercial or financial relationships that could be construed as a potential conflict of interest.

## Publisher’s Note

All claims expressed in this article are solely those of the authors and do not necessarily represent those of their affiliated organizations, or those of the publisher, the editors and the reviewers. Any product that may be evaluated in this article, or claim that may be made by its manufacturer, is not guaranteed or endorsed by the publisher.
